# Neuromodulation of innate immunity by remote ischaemic conditioning in humans: Experimental cross-over study

**DOI:** 10.1016/j.bbih.2021.100299

**Published:** 2021-07-15

**Authors:** Shaun M. May, Eric Chiang, Anna Reyes, Gladys Martir, Amour Patel, Shamir Karmali, Sanjiv Patel, Simeon West, Ana Gutierrez del Arroyo, Alexander V. Gourine, Gareth L. Ackland

**Affiliations:** a William Harvey Research Institute, Barts and the London School of Medicine and Dentistry, Queen Mary University of London, UK; b University College Hospital NHS Trust, London, UK; c Centre for Cardiovascular and Metabolic Neuroscience, Department of Neuroscience, Physiology & Pharmacology, University College London, UK

**Keywords:** Remote ischaemic conditioning, Autonomic nervous system, Vagus, Inflammation, Neutrophil, Heart rate variability

## Abstract

Experimental animal studies on the mechanisms of remote ischaemic conditioning (RIC)-induced cardioprotection against ischaemia/reperfusion injury demonstrate involvement of both neuronal and humoral pathways. Autonomic parasympathetic (vagal) pathways confer organ protection through both direct innervation and/or immunomodulation, but evidence in humans is lacking. During acute inflammation, vagal release of acetylcholine suppresses CD11b expression, a critical β2-integrin regulating neutrophil adhesion to the endothelium and transmigration to sites of injury. Here, we tested the hypothesis that RIC recruits vagal activity in humans and has an anti-inflammatory effect by reducing neutrophil CD11b expression. Participants (age:50 ​± ​19 years; 53% female) underwent ultrasound-guided injection of local anaesthetic within the brachial plexus before applying 3 ​× ​8 min cycles of brachial artery occlusion using a blood pressure cuff (RIC^block^). RIC was repeated 6 weeks later without brachial plexus block. Masked analysers quantified vagal activity (heart rate, heart rate variability (HRV)) before, and 10 ​min after, the last cycle of RIC. RR-interval increased after RIC (reduced heart rate) by 40 ​ms (95% confidence intervals (95%CI):13–66; n ​= ​17 subjects; *P* ​= ​0.003). RR-interval did not change after brachial plexus blockade (mean difference: 20 ​ms (95%CI:-11 to 50); *P* ​= ​0.19). The high-frequency component of HRV was reduced after RIC^block^, but remained unchanged after RIC (*P* ​< ​0.001), indicating that RIC preserved vagal activity. LPS-induced CD16^+^CD11b^+^ expression in whole blood (measured by flow cytometry) was reduced by RIC (3615 median fluorescence units (95%CI:475-6754); *P* = 0.026), compared with 2331 units (95%CI:-3921 to 8582); *P* = 0.726) after RIC^block^.

These data suggest that in humans RIC recruits vagal cardiac and anti-inflammatory mechanisms via ischaemia/reperfusion-induced activation of sensory nerve fibres that innervate the organ undergoing RIC.

## 1 Introduction

The bidirectional relationship between autonomic nervous and immune systems regulates cardiovascular function, metabolism, and inflammation(Carnevale and Lembo, 2021; Perrotta et al., 2018; Ziegler et al., 2018). In experimental animal models, the vagus nerve exerts cardioprotection through numerous mechanisms(Hausenloy et al., 2019; Tsutsumi et al., 2008). Vagus nerve stimulation inhibits inflammation, cytokine production and neutrophil CD11b surface expression through the cholinergic anti-inflammatory pathway(Chavan et al., 2017). Acetylcholine signalling through α7 nicotinic acetylcholine receptors (α7nAChR) inhibits release of TNFα from splenic macrophages and suppresses F-actin polymerization, the rate-limiting step for CD11*b* surface expression on circulating neutrophils(Huston et al., 2009). CD11b is a critical β2-integrin regulating neutrophil adhesion to the endothelium and transmigration to sites of injury(Fagerholm et al., 2006). Modulation of leukocyte trafficking via cholinergic signaling thus suppresses the excessive accumulation of neutrophils at inflammatory sites(Huston et al., 2009).

Vagal parasympathetic activity is critical for remote ischaemic conditioning (RIC), which protects organs from the sequelae of acute ischaemia/reperfusion injury (Mastitskaya et al., 2012) and preserves exercise capacity after myocardial infarction in rats (Machhada et al., 2020) through time-limited, repetitive ischaemia-reperfusion applied to a distant organ or tissue (Heusch et al., 2015; Walsh et al., 2007) Vagal blockade prevents RIC from reducing the extent of injury after myocardial infarction(Mastitskaya et al., 2012).

Activation of a vago-splenic axis is causally involved in RIC mediated organ protection in rats and pigs (Lieder et al., 2018), suggesting that RIC shares a common neural pathway with the cardioprotective cholinergic anti-inflammatory (Wu et al., 2017). Whilst experimental activation of the vagal anti-inflammatory pathway using specific vagus nerve-stimulating devices, non-invasive approaches to activate this pathway in humans that can be applied at scale are lacking. Laboratory data demonstrate that RIC-induced cardioprotection requires intact afferent sensory innervation of the remote organ undergoing RIC(Basalay et al., 2012; Jones et al., 2009). Therefore, in this study we tested the hypothesis that nerve blockade at the level of the brachial plexus prevents cardiac vagal and/or anti-inflammatory effects of RIC in humans. We provide the first proof-of-concept data in humans showing that RIC activates cardiac and anti-inflammatory vagal activity via a neuronal pathway.

## 2 Methods

### 2.1 Study design

We conducted an experimental cross-over study in subjects scheduled for upper limb surgery at University College London Hospitals National Health Service (NHS) Trust. The study was conducted in accordance with the principles of the Declaration of Helsinki and approved by the London (Stanmore) Research Ethics Committee (16/LO/0634) and registered on Research Registry (study 6482; January 21, 2021). Subjects provided written informed consent to participate, having given verbal consent at least 72h before the first intervention.

### 2.2 Inclusion/exclusion criteria

Subjects aged >18 years old scheduled for elective upper limb surgery requiring regional anaesthesia alone were eligible. Exclusion criteria were the presence of upper limb pathology precluding use of a blood pressure cuff, splenectomy, a history of cardiac arrythmias and/or abnormal preoperative electrocardiogram (conduction abnormality-bundle branch block, bradyarrhythmia), allergy to local anaesthetic agents, and failure to achieve loss of motor power after ultrasound guided delivery of a local anaesthetic.

### 2.3 Protocol

**Fig. 1:**
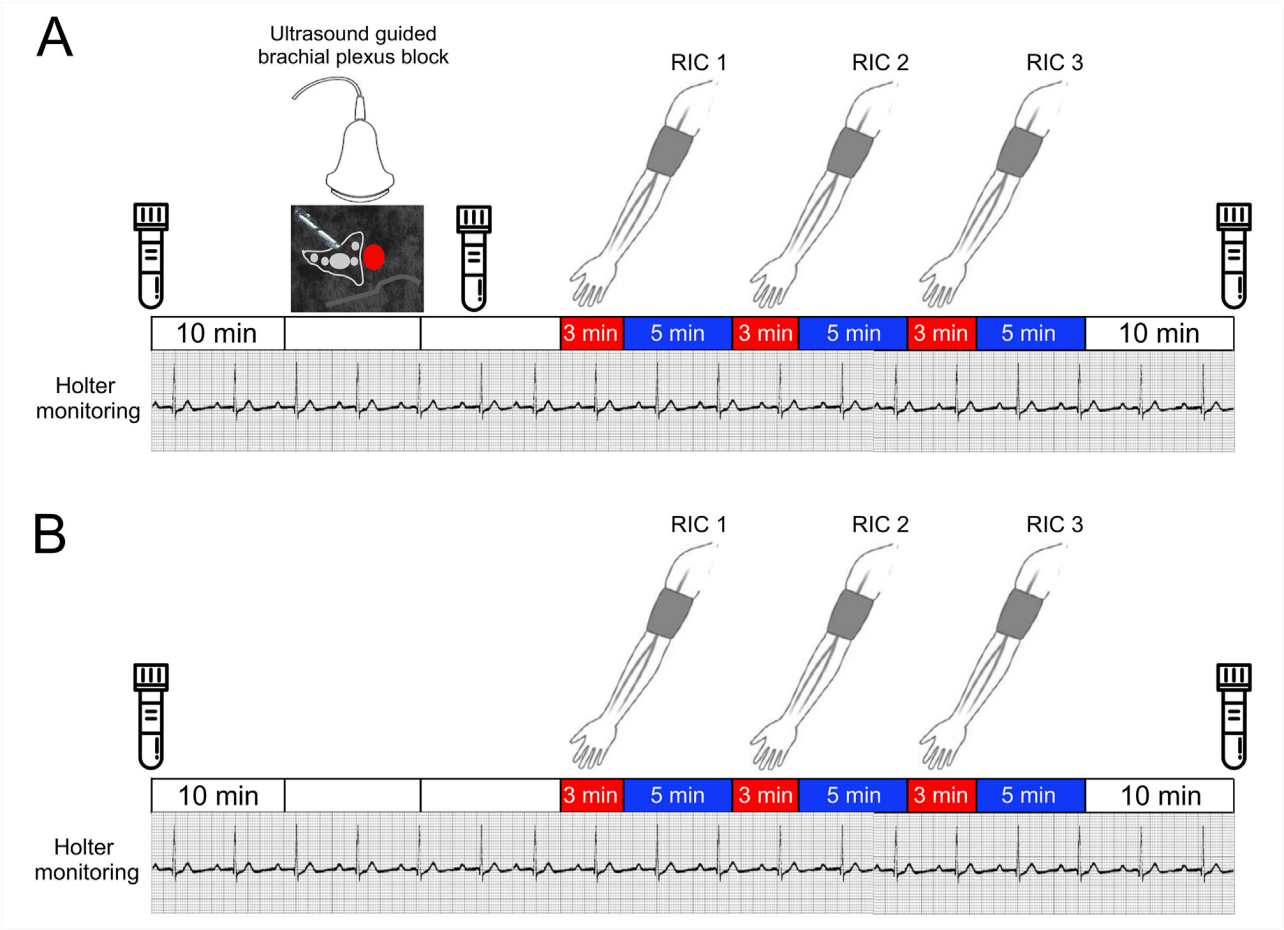
Experimental designSequence of experimental procedures for RIC undertaken in presence and absence of brachial plexus block. 10 ​min recording sessions are indicated by clear bars. Three cycles of ischaemia (applied for 3 ​min) followed by 5 ​min of reperfusion were applied. Blood samples were obtained at the times indicated by the collection bottles. A. For the first visit, brachial plexus block with ultrasound guided injection of local anaesthetic was undertaken. B. For the return visit, a time-matched protocol was followed without brachial plexus block before three cycles of RIC were undertaken.

Subjects first underwent RIC after brachial plexus block was established, prior to upper limb surgery (abbreviation RIC^block^ used in the text; Fig. 1A). On return to hospital for outpatient follow-up > 5 weeks after the surgery, RIC was repeated in the absence of brachial plexus analgesia over the same timeframe (Fig. 1B).

### 2.4 Ultrasound-guided supraclavicular brachial plexus block

All ultrasound-guided nerve blocks were conducted by senior anaesthetic staff before the surgery in a quiet dedicated procedure room (Fig. 1A). Participants were positioned in bed at 45° degrees head-up, with the head turned 45° to the non-operative side. Standard cardiorespiratory monitoring using Holter monitors were applied. Peripheral intravenous access was established but no sedation was administered. Under sterile conditions, a high-frequency linear array transducer (13–6 ​MHz, SonoSite S-Nerve; SonoSite) probe was placed over the supraclavicular fossa, parallel to the clavicle to obtain a short-axis view of the divisions of the brachial plexus and the subclavian artery, lying on the first rib. Following skin infiltration with lidocaine (1%), a 22-gauge 50 ​mm insulated echogenic needle (Pajunk, Geisingen, Germany) was guided in-plane with the ultrasound beam until the needle tip was positioned at the junction of the first rib and subclavian artery; 20 ​ml 0.5% bupivacaine was then administered using a single-injection technique, with intermittent aspiration under constant ultrasound visualisation. If paraesthesia developed, the needle tip was repositioned. After completion of the block, patients remained fully monitored until their transfer to the operating room. Assessments of sensory and motor blocks were performed by one of the investigators (SK, EC, SM, GM, AR) every 5 ​min after local anaesthetic injection. Inability of the anaesthetised arm to overcome gravity was required prior to the application of RIC.

### 2.5 Upper limb remote ischaemic conditioning

Three cycles of ischaemia/reperfusion were performed, by inflating a 12 ​cm wide blood pressure cuff over the upper arm for 3 ​min above the systolic blood pressure to occlude the brachial artery before cuff deflation for 5 ​min (Fig. 1).

### 2.6 ECG analysis

Three lead electrocardiogram recordings were captured using Lifecard CF digital Holter monitors (Spacelabs Healthcare), with a sampling rate of 1024 ​Hz. R-R intervals from ECG data were analysed after data cleaning. Heart rate variability (HRV) analysis was performed from the final 5 ​min segments within each recording periods by investigators, masked to the intervention (Fig. 1), in accord with Taskforce guidelines(Electrophysiology, 1996). Serial changes in HRV were quantified using three established measures, including time domain, frequency domain, and non-linear analyses (Kubios HRV Premium software, Version 3.1.0) (Tarvainen et al., 2014), as described previously(May et al., 2019). For time-domain measures, RR interval, minimum and maximum heart rates were analysed. We also performed spectral analysis using the parametric autoregressive method (AR) because it produces a spectrum with better resolution when short data frames are used and the spectrum can be divided into independent components(Weise et al., 1987). High frequency (0.15–0.4 ​Hz) power spectral component of HRV (AR spectrum), was used as a measure of cardiac vagal activity(Electrophysiology, 1996). Low frequency (0.04–0.15Hz) power spectral component was assessed as a measure of arterial baroreflex sensitivity(Goldstein et al., 2011). We also examined detrended fluctuation non-linear analysis correlation measures, in which a series of RR intervals are integrated and are divided into a series of regular intervals. For each interval, the fluctuation of the data from a straight line of linear interpolation was calculated. We examined changes in the short-term fractal scaling exponent (DFAα1) that quantifies the regularity of the heart rate; DFAα1values increase with pharmacological (atropine) vagal blockade and decrease with sympathetic blockade(Millar et al., 2010).

### 2.7 Flow cytometry

Blood samples were collected from patients who consented to repeat blood draws in BD Vacutainer® sodium citrate tubes (Becton Dickinson) before the first 10-min period of heart rate recording and at the end of the final 10-min period of heart rate recording. To establish that similar levels of systemic inflammation were present in each participant before each experiment, we quantified HLA-DR expression in monocytes, which is amplified by exogenous or endogenously TNF produced under the influence of IFN-γ (Arenzana-Seisdedos et al., 1988) and IL-1 (Krakauer and Oppenheim, 1993). To determine whether vagal activation by RIC may modulate acute inflammation, we examined whether the presence, or absence of brachial plexus block altered myeloid (neutrophil, monocyte) cell activation after the incubation with lipopolysaccharide (LPS). Samples were incubated with low dose of LPS (10 ƞg ml^−1^; *Escherichia coli* endotoxin, serotype O111:B4, Sigma) at 37 ​°C for 2 ​h with gentle agitation. Blood samples were immediately stained as described in Supplementary data (including gating strategy and antibodies used). Data analysis was performed by investigators masked to the intervention. Neutrophils and monocytes were identified using forward and side scatter characteristics (Supplementary figure 1), in combination with specific cell surface antibody staining for CD16 (clone VEP13), CD14 (clone TUK4), respectively (Miltenyi Biotec, Germany). Co-expression of surface CD11b, CD182 [CXCR2], HLA-DR (Miltenyi Biotec, Germany) were quantified, using frequency-minus-one and appropriate isotype controls. Acquired data (BD LSRFortessa) were analysed using FlowJo (BD Biosciences, Oxford, UK) software.

### 2.8 Statistical analysis

Manual and automated validation checks of data were undertaken. Categorical data are summarised as percentage values. Continuous data are presented as means (SD), or median [IQR], unless stated otherwise. Repeated-measures analysis of variance was used to compare heart rate and HRV measures before and after RIC, taking into account each subject, timepoint and intervention (RIC with/without nerve block). Individual comparisons between groups were calculated using post-hoc Tukey-Kramer tests. Statistical analyses were undertaken using NCSS 2020(Kaysville, UT, USA).

### 2.9 Sample size calculation

Assuming a mean resting heart rate 80 ​± ​10 bpm, we estimated that a 10 bpm difference (SD:10bpm) between RIC with versus without upper limb block would require at least 15 individuals to be recruited (α ​= ​0.01; 1-β ​= ​0.9). Allowing for 25% drop-out rate (including failure to re-attend for the follow-up protocol), the estimated sample size was initially increased to 30 subjects, but revised to 20 subjects having established >90% follow-up rate from the first 10 participants.

## 3 Results

### 3.1 Subject characteristics

**Fig. 2:**
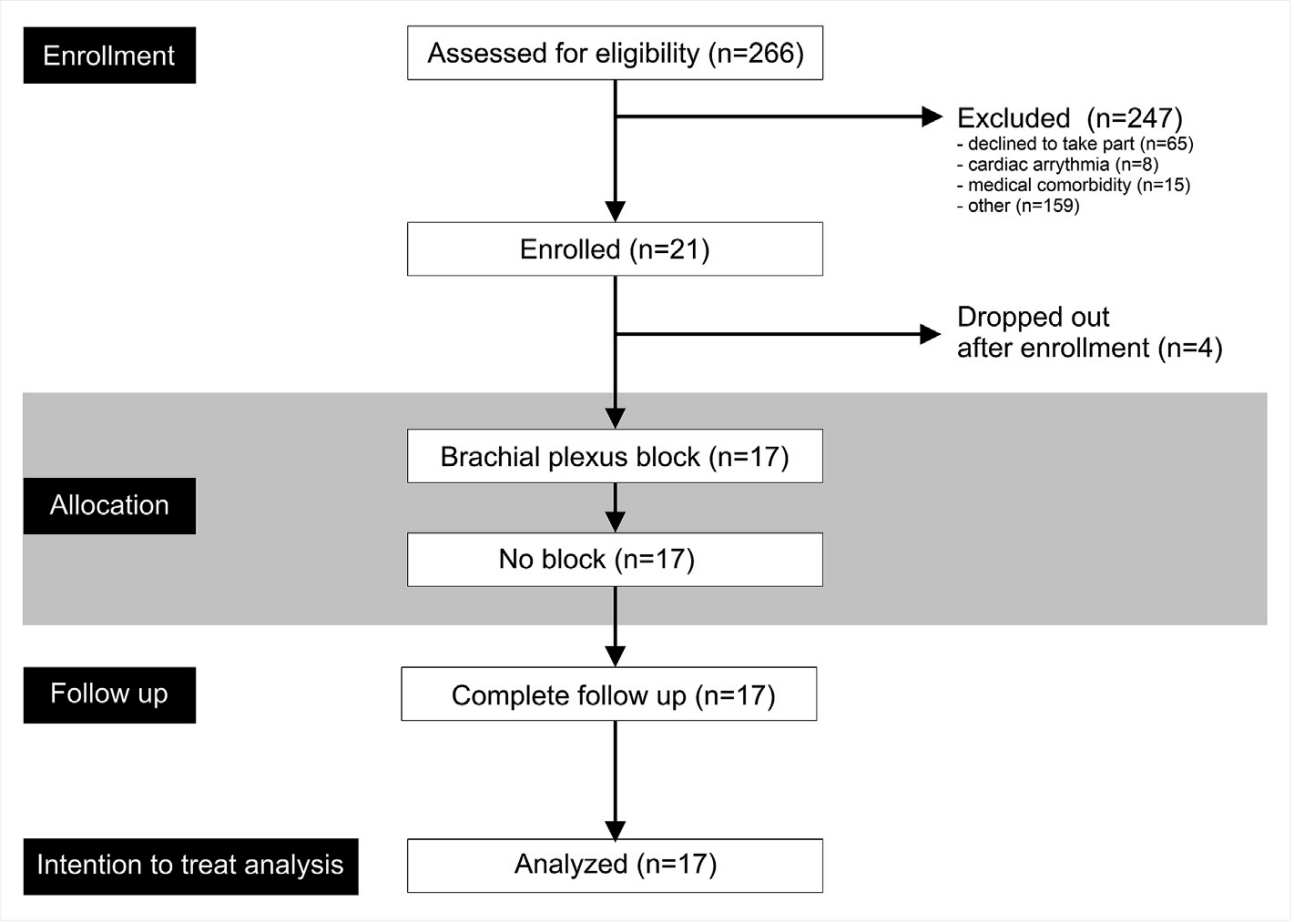
CONSORT diagram. Screening, enrolment and follow up data for study, in accord with CONSORT requirements.

A minority of the 17 participants were taking regular cardiovascular medications (Fig. 2). None had a clinical history of ischaemic heart disease. The single subject requiring oral medication for diabetic control did not have any clinical evidence for pre-existing neuropathy (Table 1). Most subjects were undergoing elective upper limb surgery for long-standing orthopaedic indications, rather than inflammatory arthropathy.

**Table 1:** Subject characteristics Data is presented as mean with standard deviations (SD) for parametric data and as median (25th-75th interquartile range) for non-parametric data. Frequencies are presented with percentages (%). Age is rounded to the nearest year. ACE-I: Angiotensin converting enzyme inhibitor. ARB: angiotensin receptor blocker.

Age (y)	56 (36–61)
Female sex (n; %)	9 (52.9%)
**Co-morbidities (n; %)**	
Asthma	5 (29.4%)
Atherosclerotic cardiovascular disease	0
Diabetes mellitus	1 (5.9%)
Current smoker	4 (23.5%)
Hypertension	2 (11.8%)
Active cancer	0 (0%)
Inflammatory arthritis	4 (23.5%)
**At least one cardiovascular medication (n; %)**	4 (23.5%)
Beta blocker	1 (5.9%)
Calcium channel blocker	1 (5.9%)
Diuretic	1 (5.9%)
Statin	1 (5.9%)
ARB/ACE-I	0
Nitrate	1 (5.9%)
NSAIDs	2 (11.8%)
Opioids	1 (5.9%)
Antidepressant	2 (11.8%)
Steroid/immunosuppresant	0

### 3.2 Effect of RIC on heart rate

**Fig. 3:**
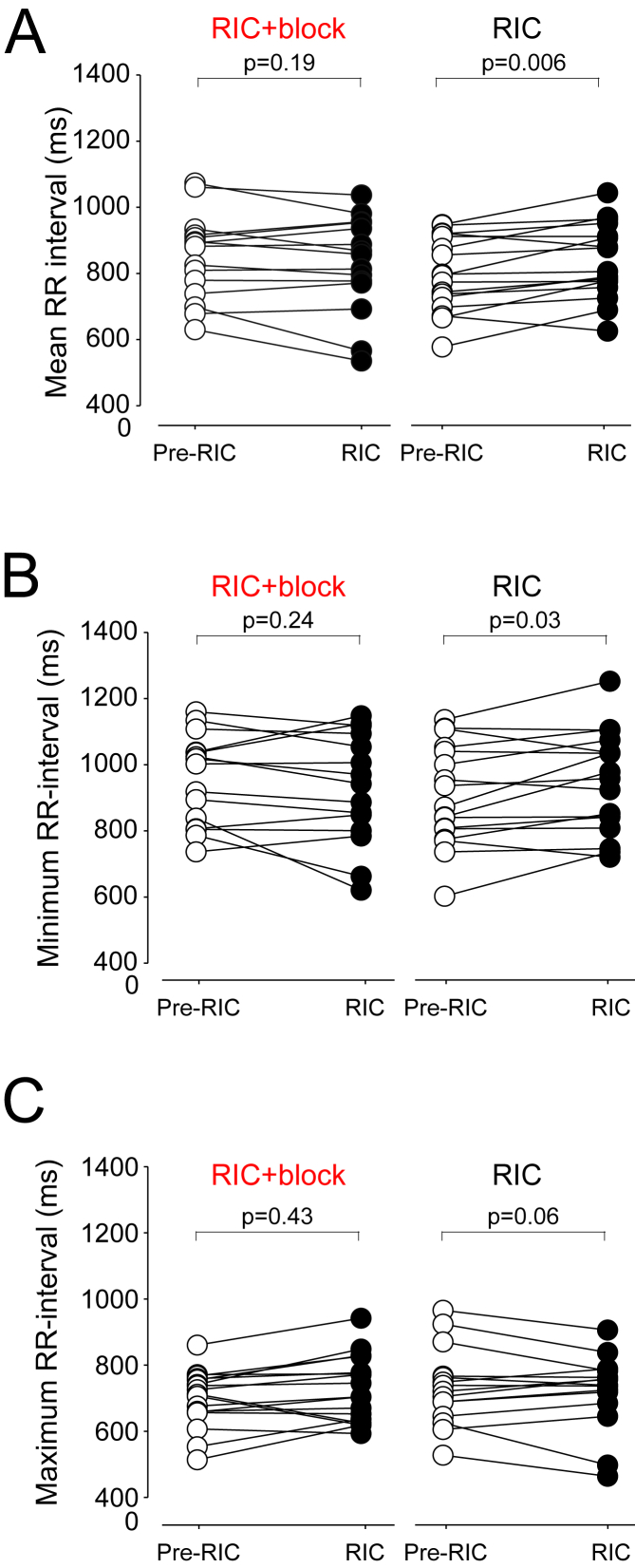
Heart rate changes before and after RIC in same individuals with/without brachial plexus block. Pre-data point for each experiment refers to final 5 ​min of data recorded within 10 ​min period before brachial plexus block was performed. Post refers to final 5 ​min of data recorded within 10 ​min time period following the final 5 ​min washout period of the third cycle of RIC. A. Individual datapoints for mean R-R interval in the presence, and absence, of brachial plexus block, during pre and post-RIC recording periods (n ​= ​17 participants). B. Individual datapoints for minimum heart rate achieved in the presence, and absence, of brachial plexus block, during pre and post-RIC recording periods., p values comparing pre versus post values (repeated-measures analysis of variance), post-hoc Tukey-Kramer test. C. Individual datapoints for maximum heart rate achieved in the presence, and absence, of brachial plexus block, during pre and post-RIC recording periods. p values comparing pre versus post values (repeated-measures analysis of variance), post-hoc Tukey-Kramer test.

RR-interval did not change after RIC in conditions of brachial plexus blockade (P ​= ​0.19). RIC increased RR-interval (i.e. slowed heart rate) by 40 ​ms (95% confidence intervals (95%CI):13–66; (Fig. 3A). In the presence of brachial plexus block, minimum RR-interval after RIC did not change (P ​= ​0.24; Fig. 3B). RIC increased minimum RR-interval by 39 ​ms (95% CI:3–74); P ​= ​0.03). Similarly, after RIC^block^, maximum RR-interval remained unchanged (P ​= ​0.43) (Fig. 3C). Maximum RR-interval increased after RIC by 28 ​ms (95% CI: 2 to 58); P ​= ​0.06). Other time-domain measures of HRV were similar between RIC and RIC^block^ (Supplementary fig. 2).

### 3.3 Effect if RIC on frequency-domain measures of heart rate variability

**Fig. 4:**
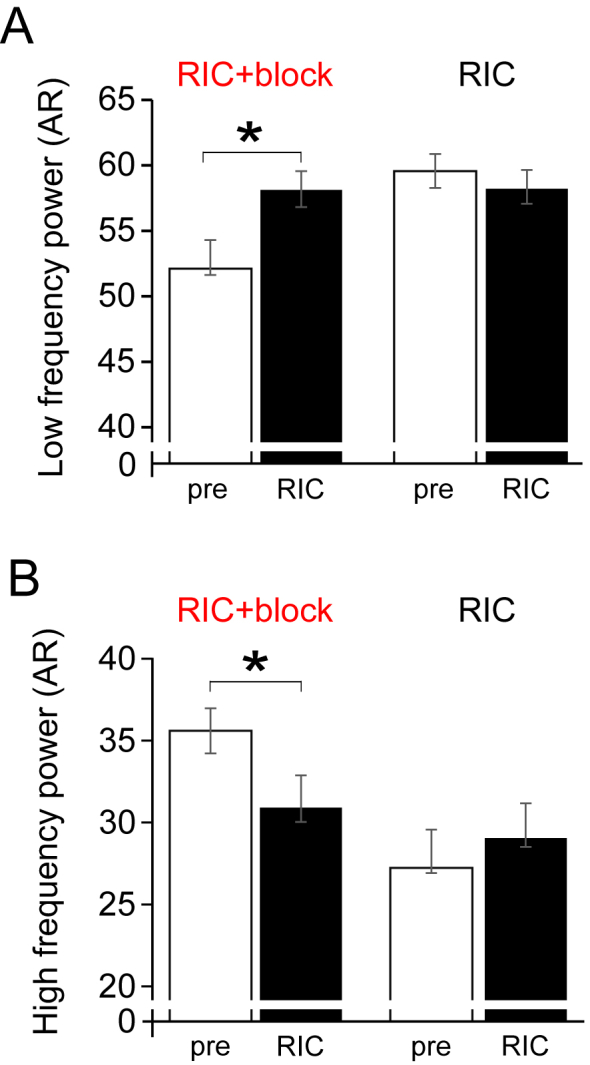
Frequency domain measures before and after RIC in same individuals with/without brachial plexus block.A. Low frequency power. B. High frequency power. Mean (95% confidence intervals) shown; values are shown for post-hoc Tukey-Kramer testing, following repeat-measures ANOVA (n ​= ​17 participants).

Low frequency (LF) band peak frequency (autoregressive spectrum) did not change after RIC, but in the presence of brachial plexus block, RIC increased LF component of HRV (Fig. 4A). Baseline HF was not different between brachial plexus block and control experiments (mean difference: 216 ​ms^2^ (95% confidence intervals: -1254 to 1685); p ​= ​0.77).

In the absence of neural block, RIC had no effect on the high-frequency (HF) component of HRV (Fig. 4B). However, in the presence of brachial plexus block, HF component was reduced in response to RIC (intervention x timepoint: *F*_(3,3.26)_ P ​= ​0.03).

### 3.4 Non-linear analysis of heart rate variability

**Fig. 5:**
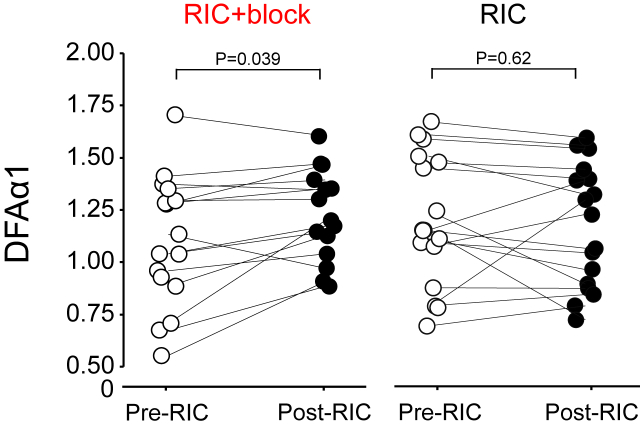
Non-linear analysis of HRV before and after RIC in same individuals with/without brachial plexus block. Individual datapoints for DFA in the presence, and absence, of brachial plexus block, pre and post RIC. p values refer to posthoc Tukey-Kramer testing, following repeat-measures ANOVA (n ​= ​17 participants).

The detrended fluctuation analysis measure DFAα1 increased after RIC was performed following brachial plexus blockade (0.124 (95% confidence intervals:0.01–0.24); P ​= ​0.039), indicative of reduced vagal tone. In the absence of brachial plexus block, DFAα1 remained unchanged after RIC (Fig. 5).

### 3.5 Effect of RIC on neutrophil activation

**Fig. 6:**
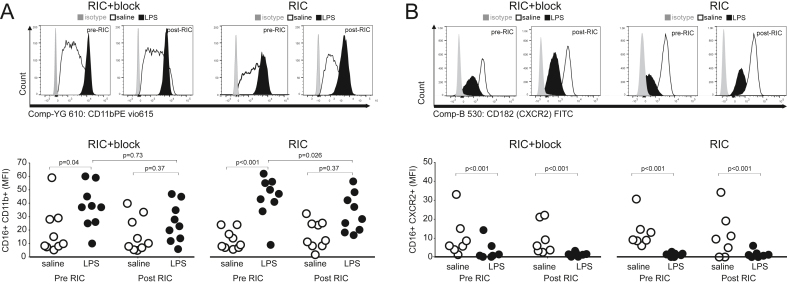
Flow cytometry analysis of surface markers of neutrophil activation before and after RIC in individuals with/without brachial plexus block. A. Surface expression of CD11b on circulating CD16 neutrophils. B. Surface expression of CXCR2 (CD182) on circulating CD16 neutrophils. P values are shown for post-hoc Tukey-Kramer testing (RIC/pre RIC versus LPS/saline control).

Repeat blood samples were obtained from nine patients who consented to repeat sampling. Systemic inflammation, as reflected by monocyte HLA-DR, was similar between each experiment before RIPC was undertaken (mean difference in HLA-DR MFI:5 (95%CI:-23 to 32); P ​= ​0.70). In blood samples collected before establishing brachial plexus blockade (Fig. 6A), expression of CD11b on neutrophils increased after the incubation with LPS (mean difference in median fluorescence intensity (MFI): 19 (95%CI:9-28); P ​= ​0.001). In blood samples collected after establishing brachial plexus blockade, but before RIC, expression of CD11b on neutrophils was unaltered (P ​= ​0.72; Supplementary fig. 3). RIC applied in conditions of brachial plexus blockade had no effect on the magnitude of LPS-induced increases in CD11b expression (mean difference in MFI: 9 (95%CI:-1 to 19; P ​= ​0.08).

When subjects returned six weeks later to undergo RIC alone, LPS increased surface expression (median fluorescence intensity) of CD11b on CD16^+^ neutrophils by 31 units (95%CI:25-38); P ​< ​0.001; Fig. 6A). RIC had no effect on unstimulated CD16^+^CD11b^+^ expression (mean difference: 3 (95%CI:-10 to 3); P ​= ​0.41). However, in blood samples collected after RIC, the effect of LPS on surface expression of CD11b in neutrophils was reduced, compared to the effect of LPS before the application of RIC (mean difference:12 arbitrary units (95%CI:5-18); P ​= ​0.002). There was no effect of RIC in the presence or absence of brachial plexus blockade on LPS-induced changes in CXCR2 expression in neutrophils (Fig. 6B).

### 3.6 Effect of RIC on monocyte activation

**Fig. 7:**
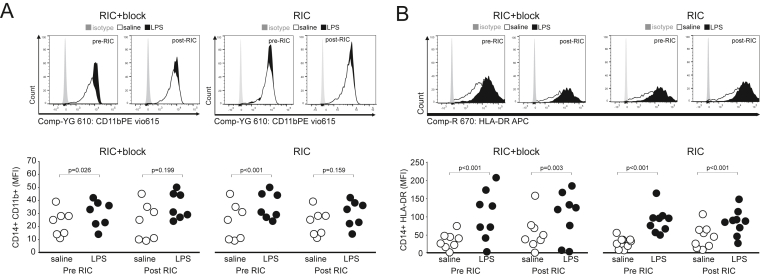
Flow cytometry analysis of surface markers of monocyte activation before and after RIC in individuals with/without brachial plexus block. A. Surface expression of CD11b on circulating CD14 monocytes. B. Surface expression of HLA-DR on circulating CD14 monocytes, which were similar at baseline in each individual between experiments (block versus control). P values are shown for post-hoc Tukey-Kramer testing (RIC/pre RIC versus LPS/saline control).

In blood samples collected before establishing brachial plexus blockade, LPS increased surface expression of CD11b on CD14^+^ monocytes (mean difference:9 (95%CI:1-18); P ​= ​0.04; Fig. 7A). RIC had no effect on CD14^+^CD11b expression (mean difference: 1 (95%CI:-25 to 23); P ​= ​0.98). In blood samples collected after RIC in conditions of brachial plexus blockade, LPS had no effect on surface expression of CD11b on monocytes (mean difference: 7 arbitrary units (95%CI:-30 to 17); P ​= ​0.73).

When the same subjects returned six weeks later, LPS increased surface expression of CD11b on monocytes (by 17 arbitrary units (95%CI:7-28); P ​= ​0.001; Fig. 7A). In blood samples collected after RIC, LPS failed to increase surface expression of CD11b (mean difference: 5 (95%CI:-16 to 5); P ​= ​0.38). There was no effect of RIC in the presence of absence of brachial plexus blockade on LPS-induced changes in HLA-DR expression on CD14^+^ monocytes (Fig. 7B).

## 4 Discussion

The results of this first-in-man study demonstrate that in humans RIC recruits cardiac vagal activity through afferent signalling from the organ/tissue undergoing RIC. When neural transmission from the peripheral ischaemic tissue was prevented by brachial plexus block using local anaesthesia, RIC had no effect on heart rate. The data also show that RIC has a rapid anti-inflammatory effect, as evident from a reduced response of neutrophils isolated from the blood collected from patients following RIC. This anti-inflammatory effect of RIC was partially abolished in conditions of brachial plexus block. Collectively the reported data suggest that RIC activates cardiac and anti-inflammatory vagus nerve activity in humans.

A role for afferent transmission in mediating RIC has been suggested in experimental models in which peripheral nociception induced by skin incisions on the abdomen established cardioprotection in mice(Jones et al., 2009). It was also found that topical application of 0.1% capsaicin (to stimulate C-fibre afferents) cream on the abdomen or paw before myocardial ischaemia/reperfusion (Redington et al., 2012) (Basalay et al., 2012) effectively reduced ischaemic damage in mice and rats via afferent signaling involving spinal nerves, sympathetic nerves, and activation of PKCε in the heart (Jones et al., 2009). Similarly, chronic neuropathic pain activates neurons within the anterior nucleus of paraventricular thalamus, which increases release of acetylcholine via the vagus nerve activity to trigger PKCε-mediated cardioprotection following ischaemia-reperfusion injury(Cheng et al., 2017). Pharmacological inhibition of extracellular signal-regulated kinase activation in the PVA abolishes neuropathic pain-induced cardioprotection, whereas pharmacologic or optogenetic activation of PVA neurons confers cardioprotection(Cheng et al., 2017). By extension, patients with established peripheral neuropathy (e.g. those with diabetes mellitus) (Jensen et al., 2012; Tang et al., 2020), or who acquire loss of vagal activity as a result of general anaesthesia, for example (May et al., 2019; Wang et al., 2004), are unlikely to benefit from RIC.

We used three measures of heart rate variability to evaluate the autonomic components, contributing to mediating the RIC effect. The 10 ​min recording period at the start and end of each protocol enabled sufficient time to capture high quality ECG data for the analysis. Frequency domain and non-linear analyses strongly suggested that parasympathetic activation is a key feature of RIC, being independent of underlying changes in heart rate(Monfredi et al., 2014). We performed spectral analysis using the parametric autoregressive method (AR) because it produces a spectrum with superior resolution using shorter data frames(Dantas et al., 2012). We used HF as an index of vagal modulation of heart rate, which is abolished in conditions of muscarinic blockade(Weise et al., 1987). RIC alone preserved the high frequency power band whereas its power declined after RIC applied in the presence of brachial plexus block, suggesting neural afferent activity is required to maintain vagal activity in this settings. In support of this, the non-linear measure DFA, which most closely corresponds to short-term fluctuations, increased after RIC following brachial plexus block. Given that DFAα1 was preserved in the absence of brachial plexus block, these data suggest that RIC initiates vagal activation since DFAα1 increases with vagal blockade and decreases with sympathetic blockade(Millar et al., 2010).

The rapid effect of RIC in conferring cardioprotection – over minutes-led us to focus primarily on circulating myeloid cells. As the archetypal first responders to acute inflammation and injury, neutrophils are activated rapidly. Neutrophil depletion reduces tissue injury after myocardial ischaemia-reperfusion in both patients (Palatianos et al., 2004) and animal models of myocardial ischaemia-reperfusion injury (Litt et al., 1989) However, dysregulated, persistent and/or over-exuberant leukocyte recruitment to ischaemic tissue can fuel excessive inflammation and exacerbate further tissue injury(Margraf et al., 2019). Our findings are the first to provide human data in support of a direct link between efferent vagal activity and response of neutrophils. The surface receptor integrin CD11b/CD18 serves as a pattern recognition receptor on phagocytes, including neutrophils, to detect pathogen and damage-associated molecular patterns(Torres-Gomez et al., 2020). This has direct relevance to cardioprotection, since a specific anti-CD18 monoclonal antibody reduces polymorphonuclear cell-mediated contractile dysfunction in an in vitro model of myocardial ischaemia-reperfusion injury by limiting polymorphonuclear cell accumulation(Lefer et al., 1993).

Similar levels of systemic inflammation were present in each participant before each experiment, as reflected by monocyte HLA-DR, the expression of which is amplified by exogenous or endogenously TNF produced under the influence of IFN-y (Arenzana-Seisdedos et al., 1988) and IL-1 (Krakauer and Oppenheim, 1993). RIC did not prevent LPS-induced downregulation of CXCR2 and increased expression of monocyte HLA-DR. Our observations are consistent with laboratory models exploring the vagal anti-inflammatory reflex, which demonstrated a reduction, rather than complete suppression, of the inflammatory response to a range of DAMPs and PAMPs(Andersson and Tracey, 2012). Nicotinic cholinergic receptors provide the receptor link between myeloid cells and the autonomic nervous system (Kanashiro et al., 2020) Unstimulated neutrophils isolated from human blood express α7nAChRs and α3β4 nAChRs(Benhammou et al., 2000). Nicotine, an agonist of the α7nAChR mediated vagal anti-inflammatory effect in macrophages, reduces levels of CD11b on the surface of neutrophils in a dose-dependent manner, by suppressing F-actin polymerization, the rate-limiting step for CD11*b* surface expression(Huston et al., 2009). Vagus nerve stimulation attenuates neutrophil surface CD11b expression only in the presence of an intact and innervated spleen(Huston et al., 2009). Reduced expression of CD11b, which also serves as a receptor for complement C3b, limits cell-mediated cytotoxicity, chemotaxis and phagocytosis.

A rapid decline in the surface expression of HLA-DR is observed in monocytes obtained from patients after acute myocardial infarction(Haeusler et al., 2012). Circulating CD14^+^HLA-DR^neg/low^ monocytes secrete high levels of TNFα, IL-6, and IL-1β which promote proinflammatory immune responses; in the expansion of this monocyte population after myocardial infarction correlates with both cardiac damage and physiological function(Fraccarollo et al., 2020). However, RIC had no effect on CD14^+^HLA-DR surface expression. Similarly, expression of the chemokine receptor CXCR2, deficiency of which limits neutrophil recruitment and the extent of myocardial infarction size, was not altered by RIC in the presence or absence of brachial plexus block.

Several randomised clinical trials, particularly in elective cardiac surgery, have failed to show consistent benefits of RIC on clinical outcomes(Heusch and Rassaf, 2016). Our study provides direct human translational data in support of the laboratory findings by demonstrating that autonomic modulation contributes to the beneficial effects of RIC. Bidirectional feedback mechanisms between the heart and the brain require both neural and humoral pathways for effective RIC(Basalay et al., 2018). The frequency and ease with which the autonomic component of RIC may be disrupted is likely to account for the results in several neutral trials. For example, peripheral neuropathy in patients with diabetes mellitus and metabolic syndrome is very common (Kazamel et al., 2021). Markedly reduced vagal activity is common in individuals who are physically deconditioned(Ackland et al., 2018, 2019; Machhada et al., 2017). Furthermore, neural processing in autonomic pathways within the central nervous system is profoundly disrupted by anaesthetic agents(Jin et al., 2012; Wang, 2009).

The development of this human model enabled each subject to serve as their own control while remaining masked to laboratory outcomes. Capturing both autonomic and immunologic readouts added further complementary data to address the central hypothesis that RIC stimulates cardiac and anti-inflammatory vagal pathways. We were unable to randomise the sequence of block versus control RIC, as subjects gave consent prior to elective upper limb surgery. Similarity in both autonomic and inflammatory measures at the start of both control and block protocols suggest that the impact of randomising the sequence of interventions would not be significant. Brachial plexus nerve block is not a minor procedure for subjects who do not require surgery, hence the choice of participants were preoperative patients. It is important to highlight that ultrasound-guided brachial plexus block was undertaken without sedation, which alters autonomic function(Karmali et al., 2017). Although data were captured only during the protocol period, we have established that this model of brachial plexus block serves as a potentially useful tool over the longer term to further dissect the mechanisms of RIC in humans.

In summary, we have shown, for the first time in humans, that RIC recruits vagal cardiac and anti-inflammatory mechanisms, most likely via ischaemia/reperfusion-induced activation of sensory nerve fibres that innervate the organ or tissue undergoing RIC – the phenomenon previously identified in laboratory animal models. Closing this translational gap in mechanistic knowledge in humans explains, in part, the variable effect of RIC in conferring cardiac and renal protection(Basalay et al., 2018). Our data suggest that a personalised medicine approach may benefit individuals who are capable of mounting an integrated innate response to RIC, that involves recruitment of vagal mechanisms.

## Funding

This work was supported by the 10.13039/501100000274British Heart Foundation (Refs: RG/14/4/30736 and RG/19/5/34463); Wellcome Trust Senior research award (AVG); 10.13039/100011716British Journal of Anaesthesia/10.13039/501100001297Royal College of Anaesthetists Basic Science Career Development Award (GLA); British Oxygen Company research chair grant, Royal College of Anaesthetists (GLA); 10.13039/501100000272UK National Institute for Health Research. GLA holds a NIHR Advanced Fellowship (NIHR 300097).

## Authors' contribution statement

Shaun May: patient recruitment, Holter data capture and analysis; sample collection.

Eric Chiang: Holter data capture; flow cytometry-processing and analysis.

Anna Reyes, Gladys Martir, Amour Patel, Shamir Karmali,: patient recruitment and sample collection.

Simeon West, Sanjiv Patel: supervision/delivery of supraclavicular blocks.

Ana Gutierrez del Arroyo: flow cytometry-processing and analysis.

Alexander V. Gourine: study design, writing the manuscript.

Gareth L. Ackland: study design and oversight, Holter data capture and analysis; sample collection, writing the manuscript.

## Conflicts of interest/Competing interests

GLA- Editor, British Journal of Anaesthesia; consultancy work for GlaxoSmithKline, unrelated to this work.
