# Supplementary material for: Neuromodulation of innate immunity by remote ischaemic conditioning in humans: Experimental cross-over study
Source: Brain Behav Immun Health. 2021 Jul 15;16:100299. doi: 10.1016/j.bbih.2021.100299 (PMC8417773; doi:10.1016/j.bbih.2021.100299)

Activation of the vagal anti-inflammatory reflex by remote ischaemic conditioning in humans: experimental cross-over study.

Shaun M. May,*[1] Eric Chiang,*[1] Anna Reyes,[2] Gladys Martir,[2] Amour Patel,[1] Shamir Karmali,[1] Sanjiv Patel,[2] Simeon West,[2] Ana Gutierrez del Arroyo,[1] Alexander V. Gourine,[3] Gareth L. Ackland[1]

SUPPLEMENTARY DATA

Contents

Supplementary Figure 1. Gating strategy 2
Supplementary Figure 2. Time domain measures of HRV. 3
Supplementary Figure 3. Lack of acute effect of brachial plexus block on flow cytometry measures. . 4

Supplementary Figure 1. Gating strategy

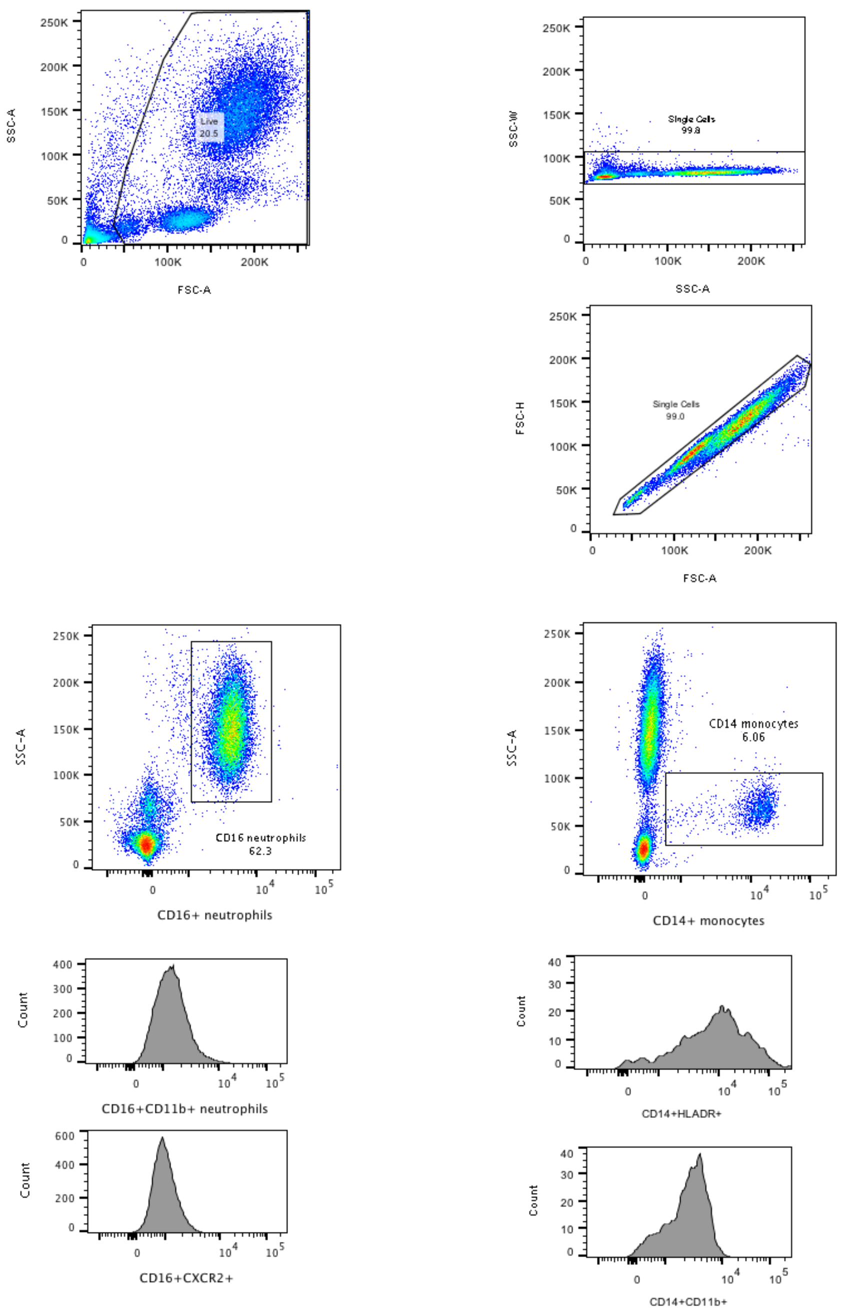

Supplementary Figure 2. Time domain measures of HRV.

A. SDNN, the standard deviation of NN intervals

B. RMSSD, Root Mean Square of the Successive Differences in RR interval.

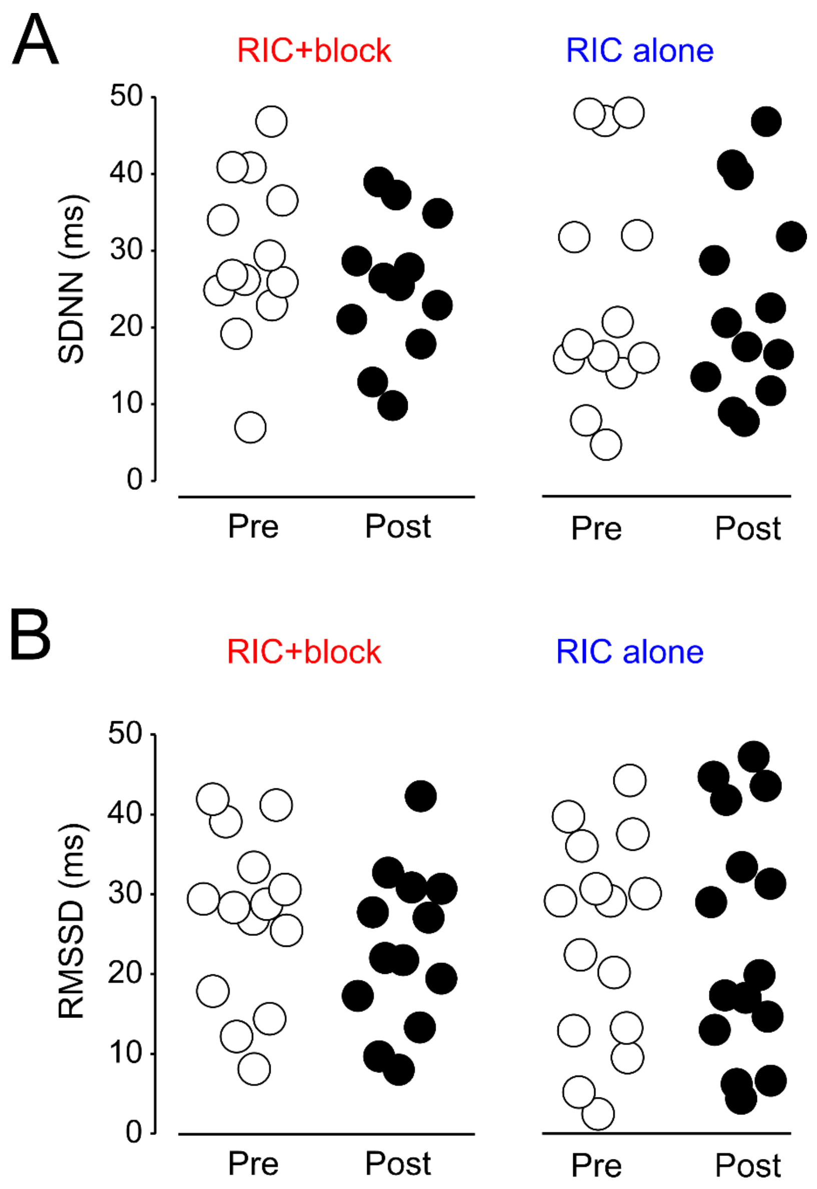

Supplementary Figure 3. Lack of acute effect of brachial plexus block on flow cytometry measures.

Representative plots from same individual before and after injection of local anaesthetic into brachial plexus.

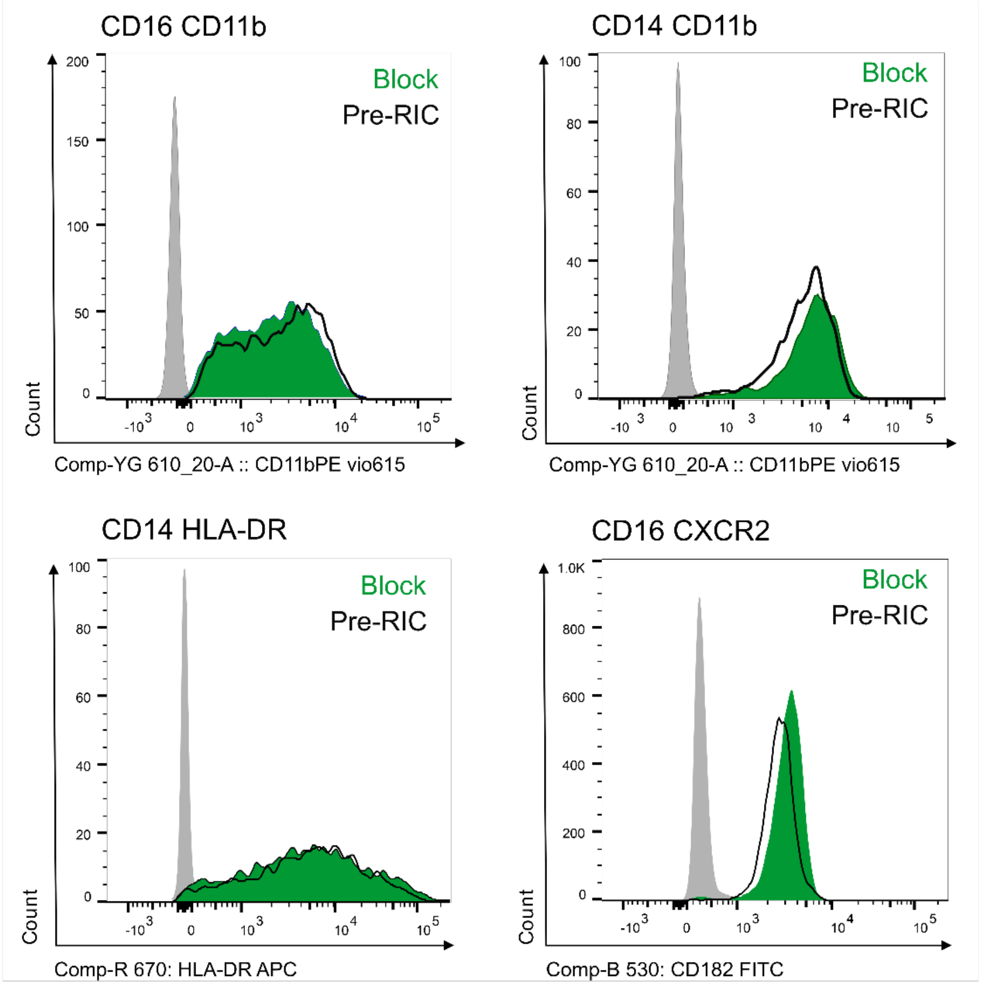

Supplement: Multimedia component 1 [file mmc1.pdf]
